# The Impact of COVID-19-Related Restrictions on the Incidence of Diaphyseal and Distal Forearm Fractures: A Retrospective Analysis

**DOI:** 10.3390/medicina62050966

**Published:** 2026-05-15

**Authors:** Katja Brabec, Nicola Stringari, Manuel Gahleitner, Paul Michael Schwarz, Sandra Feldler, Simon Kargl, Tobias Gotterbarm, Lorenz Pisecky, Matthias Holzbauer

**Affiliations:** 1 Medical Faculty, Johannes Kepler University Linz, Altenbergerstr. 69, 4040 Linz, Austria; katja.brabec@kepleruniklinikum.at (K.B.); paul.schwarz@kepleruniklinikum.at (P.M.S.); sandra.feldler@kepleruniklinikum.at (S.F.); simon.kargl@kepleruniklinikum.at (S.K.); tobias.gotterbarm@kepleruniklinikum.at (T.G.); lorenz.pisecky@kepleruniklinikum.at (L.P.); matthias.holzbauer@a1.net (M.H.); 2 Department of Pediatric Surgery, Kepler University Hospital GmbH, Krankenhausstrasse 9, 4020 Linz, Austria; nicola.stringari@kepleruniklinikum.at; 3 Department of Orthopaedics and Traumatology, Kepler University Hospital GmbH, Krankenhausstrasse 9, 4020 Linz, Austria; 4 Department of Plastic, Aesthetic and Reconstructive Surgery, Kepler University Hospital GmbH, Krankenhausstrasse 9, 4020 Linz, Austria

**Keywords:** pediatric forearm fractures, distal radius fractures, COVID-19 pandemic, injury epidemiology, pediatric trauma, lockdown restrictions, time-series analysis

## Abstract

*Background and Objectives*: Pediatric forearm fractures are among the most common childhood injuries. COVID-19-related societal restrictions, including school closures and suspension of sports activities, altered children’s daily routines and may have influenced injury patterns. This study aimed to evaluate whether periods of stricter COVID-19 restrictions were associated with changes in the incidence of pediatric distal and diaphyseal forearm fractures after accounting for seasonal variation and long-term temporal trends. *Materials and Methods*: This retrospective observational time-series study analyzed pediatric patients aged 0–17 years who underwent forearm radiography between January 2018 and June 2023 at a tertiary pediatric trauma center. Cases with radiologically confirmed distal or diaphyseal forearm fractures or epiphyseal injuries were included. Monthly fracture counts were analyzed using generalized linear models with logarithmic link functions. Exposure variables included a COVID-19 restriction index based on governmental measures and a binary pandemic indicator. Seasonal variation and long-term temporal trends were included as covariates. *Results*: A total of 5702 forearm radiographs were identified, of which 4041 trauma-related presentations met the inclusion criteria. Among these, 2014 children had confirmed forearm fractures. Boys accounted for 61% of cases, and the median age was 9 years (IQR 5). Most fractures were treated conservatively (88%). The most frequent injury mechanisms included soccer-related injuries (9.6%) and bicycle falls (7.3%). In regression analyses adjusted for seasonal variation and temporal trends, neither the COVID-19 restriction index (IRR 1.01, 95% CI 0.87–1.17; *p* = 0.95) nor the pandemic period indicator (IRR 0.99, 95% CI 0.37–2.65; *p* = 0.98) was significantly associated with monthly fracture counts. The wide confidence interval of the pandemic indicator reflects limited statistical precision and suggests that both clinically relevant decreases and increases in fracture incidence cannot be excluded. *Conclusions*: No sustained long-term changes in the incidence or injury patterns of pediatric forearm fractures were observed during the COVID-19 pandemic. Temporary fluctuations during early lockdown phases were not independently associated with governmental restrictions after adjustment for seasonal variability and long-term trends.

## 1. Introduction

Forearm fractures represent one of the most common injuries in childhood [1]. Distal radius fractures constitute the most frequent fracture type in the pediatric population [2]. According to previous reports, they account for approximately 25–43% of all fractures in children. The epidemiology and incidence of fractures vary considerably depending on geographic location, age, and sex. Therefore, reported incidence rates differ between studies. For example, Naranje et al. reported an incidence ranging from 12 to 36 fractures per 1000 children per year [3]. Pediatric patients between 10 and 14 years of age have the highest risk of sustaining fractures [3]. Boys demonstrate the highest fracture incidence at approximately 13 years of age, whereas girls show the highest incidence around 10 years of age [4]. Regarding sex distribution, boys (64%) are more frequently affected by forearm fractures than girls (36%) [1]. A seasonal variation has also been observed, with pediatric distal forearm fractures occurring more frequently during the summer months [4].

Common types of pediatric forearm fractures include torus (buckle) fractures, greenstick fractures, and complete fractures. The fracture pattern and degree of displacement depend largely on the mechanism of injury. Most pediatric forearm fractures result from indirect trauma, particularly falls [5]. Ryan et al. reported that 83% of forearm fractures were caused by falls, with falls from monkey bars representing the most frequently reported specific mechanism [6]. In addition, a Danish survey found that 39% of all distal forearm fractures were associated with sports activities [4].

At the beginning of 2020, the COVID-19 pandemic led to profound changes in the daily lives of children worldwide, affecting both personal and school-related activities [7]. Alterations in daily activity patterns resulted in shifts in the incidence and distribution of pediatric orthopedic trauma [8], as well as changes in fracture mechanisms [9]. Children and adolescents were particularly affected by curfews, the closure of educational institutions, and restrictions on leisure and sports activities. These governmental measures resulted in an overall reduction in physical activity levels [10,11,12].

Several international studies investigating pediatric injury incidence during the pandemic have reported heterogeneous findings. Comparative analyses suggest that fracture incidence declined in many settings, particularly during the early phase of the pandemic [8,13,14,15,16]. However, some studies observed only minimal changes in injury incidence, whereas others even reported slight increases in fracture numbers [15,17]. Studies specifically examining pediatric forearm fractures during the pandemic have likewise yielded variable results, ranging from minor reductions to substantial decreases in fracture incidence [8,17,18].

The aim of the present study was to evaluate whether periods characterized by stricter COVID-19-related societal restrictions were associated with a statistically significant reduction in fracture incidence, independent of seasonal variation and underlying temporal trends. To the best of our knowledge, no comparable studies investigating the incidence of pediatric forearm fractures in Austria have been published to date.

## 2. Materials and Methods

### 2.1. Study Design and Population

The regional ethics review committee approved this study (No.: EK-1324/2023). Informed consent was waived owing to the retrospective study design.

This retrospective observational time-series study analyzed distal and diaphyseal forearm fractures recorded between January 2018 and June 2023. The primary outcome variable was the monthly number of fractures.

**Figure 1:**
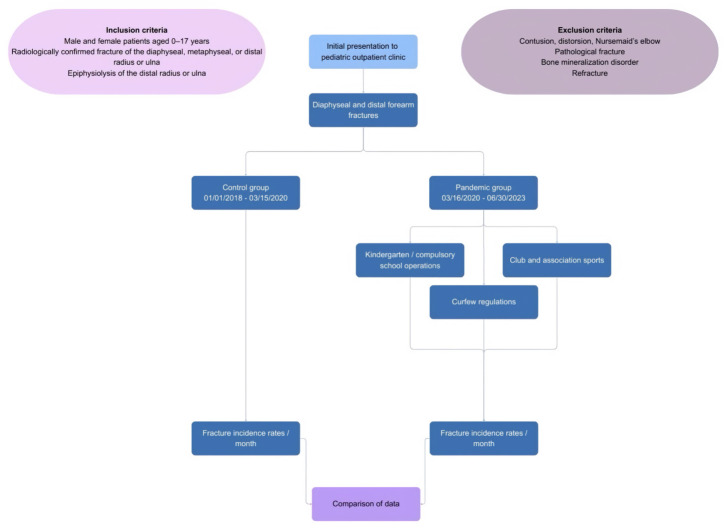
Flow chart to illustrate the study design and the distribution of cohorts.

Consecutive pediatric patients aged 0–17 years who presented to the pediatric outpatient clinic of the Department of Pediatric Surgery and the Department of Orthopedics and Traumatology and underwent forearm radiography were identified using the hospital medical information system. Patients presenting after a new traumatic event were included if radiological imaging confirmed a distal or diaphyseal fracture of the radius or ulna, or an epiphyseal injury. Patients who underwent radiography following trauma but without radiological evidence of fracture (e.g., soft-tissue injury or dislocation) were excluded from the main analysis; however, their number was recorded to reflect the overall volume of trauma-related outpatient visits. The study design is illustrated in Figure 1, which also includes detailed inclusion and exclusion criteria.

Epidemiological variables including sex, age, and cause of injury were collected. Causes of injury were categorized into predefined main categories and subcategories. All radiographs were reviewed by the lead author (KB). Cases with uncertain findings were discussed with the corresponding author (MH) until consensus was reached. A formal assessment of interobserver or intraobserver reliability was not performed.

### 2.2. Fracture Classification and COVID-19 Exposure

Fractures were classified as complete fractures, greenstick fractures, buckle (torus) fractures, bowing fractures, incomplete fractures, comminuted fractures, styloid process fractures, capsular avulsions, or epiphysiolysis according to the Salter–Harris (SH) classification (types I–V). Specific injury patterns—including Monteggia, Monteggia-like, Galeazzi, and Galeazzi-equivalent fractures—were also identified.

Fracture location was categorized as diaphyseal, metaphyseal, epiphyseal, or isolated bony avulsion. The diaphysis was defined as the bone segment between the metaphyses. To differentiate metaphysis from diaphysis, the length of the corresponding growth plate was used as a radius to draw arcs from the corners of the growth plate toward the diaphysis; the intersection of these arcs defined the metaphyseal–diaphyseal boundary. The diametaphyseal region was not defined separately. The epiphysis was defined as the most distal part of the bone and was separated from the metaphysis by the epiphyseal plate.

To assess the influence of the COVID-19 pandemic, the study period was divided into two cohorts according to the regional onset of pandemic-related restrictions. The control period comprised 1 January 2018 to 15 March 2020, whereas the pandemic period extended from 16 March 2020 to 30 June 2023, which marked the formal end of the pandemic in Austria.

Governmental COVID-19 measures were reviewed on a monthly basis and categorized according to kindergarten and primary school operations, lower and upper secondary school operations, organized club sports, and curfew regulations.

### 2.3. Statistical Methods

Statistical analyses were performed using Microsoft Excel and IBM SPSS Statistics (version 31.0.1.0). Descriptive statistics were applied. Normality of continuous variables was assessed using the Kolmogorov–Smirnov test with Lilliefors correction. Continuous variables are presented as mean ± standard deviation (SD) or median with interquartile range (IQR), depending on distribution. Ordinal variables are reported as median (IQR), and categorical variables as absolute frequencies.

Incidence rates were estimated based on the pediatric population within the catchment area of the hospital. Approximately 38% (575,997 of 1,515,781 inhabitants) of the population of Upper Austria reside in the central region of the province. Based on annual birth statistics for Upper Austria (14,797 births per year), approximately 5600 births occur annually in this region. Considering the pediatric age range of 0–17 years (18 age cohorts), the pediatric population of the catchment area was estimated at approximately 101,000 children and adolescents. These incidence estimates should be interpreted as approximations, as they are based on population assumptions and do not account for migration or treatment outside the study center. Accordingly, all following incidence values are reported as estimated rates, with primary analyses based on absolute fracture counts.

To quantify the cumulative impact of COVID-19-related societal restrictions, a composite restriction index was developed. Similar composite indices have been widely used to characterize the intensity of governmental responses during the pandemic, most notably the Oxford COVID-19 Government Response Stringency Index, which aggregates multiple policy indicators such as school closures, movement restrictions, and limitations on social interaction [19].

However, such global indices may not adequately reflect regional policy implementation and factors specifically influencing pediatric activity patterns. Therefore, a customized restriction index was constructed to capture measures particularly relevant to children and adolescents in the Austrian setting.

Four domains of governmental measures were included: (1) kindergarten and primary school operations, (2) secondary school operations, (3) organized sports activities, and (4) curfew regulations. Each domain was scored on an ordinal scale (0 = complete restriction, 1 = partial restriction, 2 = no restriction). The sum of these scores yielded a composite index ranging from 0 (maximum restrictions) to 8 (no restrictions), which was used as a proxy measure for restriction intensity over time.

To improve transparency, explicit criteria were defined for each restriction level. “Complete restriction” (score = 0) was assigned when activities were fully suspended (e.g., complete school closures, cancellation of organized sports, or full curfew restrictions). “Partial restriction” (score = 1) included hybrid schooling models, limited attendance, restricted group sizes, or time-limited curfews. “No restriction” (score = 2) was defined as unrestricted operation without relevant limitations.

For example, a nationwide school closure with distance learning was classified as complete restriction (0), whereas alternating in-person attendance or reduced class sizes were classified as partial restriction (1). Similarly, a full curfew or strict stay-at-home order was classified as complete restriction, whereas time-restricted curfews or limitations on nighttime movement were classified as partial restriction (see Table S1).

Monthly fracture counts were analyzed using generalized linear models with a logarithmic link function. An initial Poisson regression model was fitted, and overdispersion was assessed using goodness-of-fit statistics, including the deviance and Pearson chi-square statistics relative to their degrees of freedom. Moderate overdispersion was observed (Pearson χ^2^/df = 1.44; deviance/df ≈ 1.40), indicating violation of the Poisson assumption of equidispersion. Therefore, negative binomial regression models were used for all primary analyses.

Seasonal variation was modeled using calendar month as a categorical variable. Interaction terms between exposure variables (restriction index or pandemic indicator) and calendar month were included to assess potential seasonal effect modification.

Autocorrelation of residuals was evaluated using inspection of autocorrelation function (ACF) plots. No substantial residual autocorrelation was observed. Therefore, no additional autoregressive terms or robust standard error adjustments were applied.

Two alternative exposure variables were evaluated in separate models: the continuous restriction index and a binary pandemic indicator (pre-pandemic vs. pandemic period). These variables were not included simultaneously to avoid collinearity.

Seasonal variation was controlled for by including calendar month as a categorical covariate, while long-term temporal trends were modeled using a continuous variable representing months since the start of the observation period. March 2020 was excluded from regression analyses due to its mixed pre-pandemic and pandemic exposure.

Results are presented as incidence rate ratios (IRRs) with corresponding 95% confidence intervals. A two-sided *p*-value < 0.05 was considered statistically significant.

## 3. Results

### 3.1. Study Population and Demographics

The medical database identified 5702 cases in which a forearm radiograph was obtained during the study period. Of these, 4041 presentations followed a new traumatic event and were investigated using forearm radiography. Among these patients, 2014 children had radiologically confirmed distal or diaphyseal forearm fractures or epiphyseal injuries.

Demographic characteristics of children with fractures were analyzed. Boys presented more frequently with forearm fractures (*n* = 1244, 61%) than girls (*n* = 770, 38%). The median age of patients with forearm fractures was 9 years (IQR: 5 years). When stratified by sex, the median age was 9 years (IQR: 6 years) in boys and 8 years (IQR: 4 years) in girls.

Details regarding fracture localization are presented in Table 1. Of the 2014 fractures, 59 cases (2.9%) were isolated ulna fractures without involvement of the radius. The distribution of radial fracture types is presented in Table 2.

### 3.2. Treatment and Causes of Injury

Most pediatric patients with forearm fractures were treated conservatively (*n* = 1774, 88%), whereas surgical treatment, including Kirschner wire (K-wire) fixation or elastic stable intramedullary nailing (ESIN), was required in 240 patients (12%). Overall, the distribution of operative versus nonoperative treatment remained stable throughout the study period without apparent temporal shifts between the pre-pandemic and pandemic phases.

The main categories of injury causes stratified by age group are presented in Table 3. Analysis of injury subcategories revealed that the most frequent specific cause of forearm fractures was injury sustained while playing soccer (*n* = 193, 9.6%), followed by falls from a bicycle (*n* = 146, 7.3%), injuries or falls at kindergarten or school (*n* = 117, 5.8%), and injuries during school sports activities (*n* = 103, 5.1%).

Data on post-treatment complications were not systematically available due to the retrospective design and the lack of standardized follow-up documentation, and were therefore not assessed.

### 3.3. Temporal Trends and COVID-19 Restrictions

**Figure 2:**
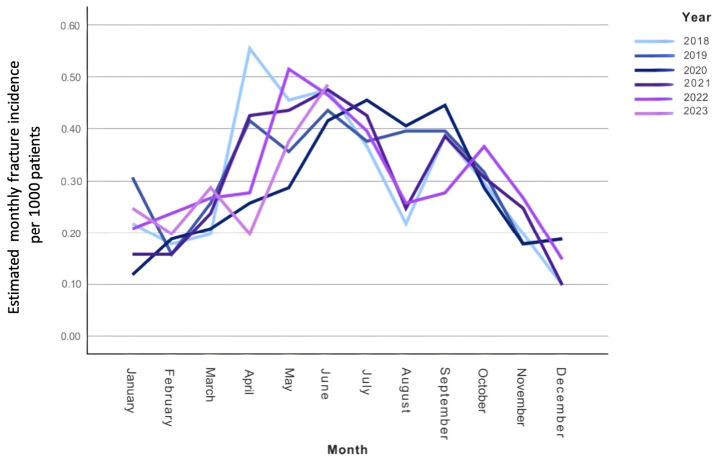
Monthly fracture incidence per 1000 patients, from 01/01/2018 to 06/30/2023. Each line graph represents a separate calendar year; the graph for 2023 ends with the evaluation period in June 2023.

**Figure 3:**
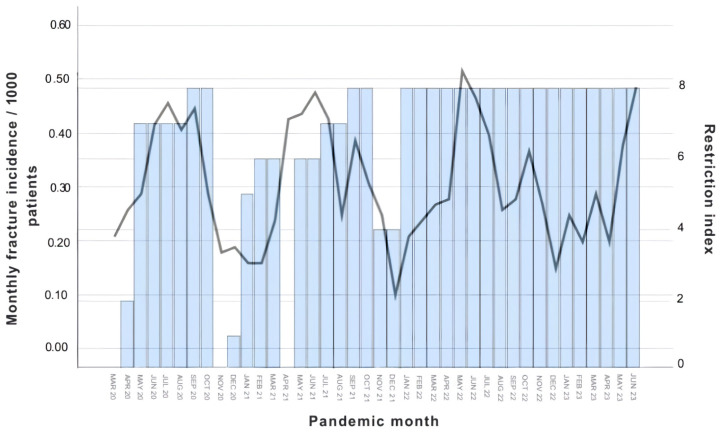
Comparison of the monthly forearm fracture incidence (line graph, fractures/1000 children) with the restriction index (bar graph, 0–8 points) in the respective pandemic months.

Estimated monthly fracture incidence is illustrated in Figure 2, while Figure 3 shows the relationship between monthly fracture incidence and the COVID-19 restriction index.

In negative binomial regression models adjusted for seasonal variation and long-term temporal trends, neither the COVID-19 restriction index (incidence rate ratio [IRR] = 1.01, 95% CI 0.87–1.17, *p* = 0.95) nor a binary pandemic indicator (IRR = 0.99, 95% CI 0.37–2.65, *p* = 0.98) was significantly associated with monthly fracture counts. The wide confidence interval of the pandemic indicator reflects limited statistical precision and suggests that both clinically relevant decreases and increases in fracture incidence cannot be excluded.

Seasonal variation was controlled for by including calendar month as a categorical variable. Although higher fracture counts were observed during spring and summer months, none of the month-specific effects reached statistical significance (all *p* > 0.18), indicating no statistically detectable seasonal variation after adjustment for covariates. Furthermore, interaction terms between exposure variables and calendar month were not statistically significant (restriction index × month: *p* = 0.84; pandemic × month: *p* = 0.91), indicating no evidence of seasonal effect modification. Furthermore, no independent long-term temporal trend was detected (IRR = 1.00, 95% CI 0.98–1.03, *p* = 0.98).

## 4. Discussion

The main finding of this study is that no evidence was identified for a sustained change in the incidence of pediatric forearm fractures during the COVID-19 pandemic. Although fracture numbers declined during the early lockdown phases, this reduction was not independently associated with pandemic-related restrictions after adjustment for seasonal variation and long-term temporal trends. Overall, the results indicate that the COVID-19 pandemic did not lead to a long-term structural shift in the epidemiology of pediatric forearm fractures within the study catchment area.

Several international studies have reported a reduction in overall pediatric fracture incidence during periods of pandemic-related restrictions, including, in some cases, forearm fractures specifically. These reductions were most pronounced during the early months of 2020, corresponding to the initial phase of the pandemic [8,13,14,15,16,20]. Markiewitz et al. reported a 27% decrease in monthly pediatric fractures, persisting into the latter half of 2020 [8], while Bram et al. described a 2.5-fold reduction in pediatric fractures during the pandemic [13]. Similarly, Turgut et al. observed a one-third reduction in fracture incidence across both pediatric and adult populations [14]. Park et al. demonstrated significant declines in both prevalence (34,626 to 24,789, *p* < 0.001) and incidence (29,804 to 18,898, *p* < 0.001) of pediatric fractures [16], and Lapsa et al. reported reductions of 40% compared to 2019 and 28% compared to 2018 [20]. These findings have been described across diverse healthcare settings, including the United States [8,13,20], Turkey [14], and Korea [16]. In many of these studies, fracture incidence returned to pre-pandemic levels following the relaxation of restrictions and resumption of regular activities [8,15].

In contrast, other investigations have reported minimal or no significant changes in pediatric fracture incidence during the pandemic [17,18]. David et al. observed no relevant differences in incidence or demographic characteristics of pediatric forearm fractures in a Romanian tertiary care center, with a reduction of less than 5% [17]. Similarly, Olech et al. reported only a modest decrease of 3.5% in distal radius fractures in a Polish cohort [18], and Schultz et al. found stable incidence rates of pediatric elbow fractures in a single-center study in the United States [21]. The findings of the present study align with this latter group of studies, demonstrating only transient reductions during early lockdown phases without a sustained effect. Importantly, the present analysis extends prior work by incorporating adjustments for both seasonal variation and long-term temporal trends. After accounting for these factors, the observed decline in fracture numbers during the early pandemic phases was not statistically associated with COVID-19 restrictions. These findings may indicate that previously reported reductions—depending on the methodological approach of the respective study—may have been influenced by underlying seasonal patterns or temporal variability.

Potential interaction effects, such as a modification of restriction effects by season, were not included in the model. Given the limited number of observations, inclusion of interaction terms would have increased the risk of overfitting and reduced model stability.

Seasonal variation in pediatric fracture incidence is well established and is commonly attributed to increased outdoor activity and sports participation during warmer months [22,23,24,25]. Consistent with this, the present study demonstrated higher fracture counts during spring and summer. These recurring patterns underscore the necessity of accounting for seasonality in epidemiological analyses of injury incidence over extended time periods.

With respect to injury mechanisms, falls were the predominant cause of fractures, in line with prior epidemiological studies of pediatric forearm injuries [4,5,6]. The most frequent specific mechanisms included falls related to transportation, particularly bicycle use, as well as injuries sustained on playgrounds, in domestic environments, and during ball sports such as soccer. These findings reflect typical activity patterns among children and adolescents and emphasize the close association between recreational behavior and injury risk [4,25,26]. Variations in the most common sports-related injury mechanisms across studies likely reflect regional differences in sports participation. For example, fractures in the United States are more commonly associated with football and basketball [27,28], whereas in European populations, including Austria and Switzerland, soccer-related injuries predominate [29,30]. In the present study, no formal statistical testing was performed to compare injury mechanisms between pre-pandemic and pandemic periods. Therefore, these observations should be interpreted with caution and regarded as exploratory.

The COVID-19 pandemic markedly altered children’s daily routines through school closures, suspension of organized sports, and reduced social interactions [10,11]. These changes were reflected in temporary shifts in injury mechanisms observed in the present study. During periods of school closure, fewer fractures occurred in educational settings, while a relatively higher proportion of injuries were associated with activities at home, outdoor leisure, and individual transportation such as cycling. Similarly, injuries related to organized sports declined during phases when club-based activities were restricted. During periods of strict curfew, a greater proportion of fractures occurred in domestic environments. Comparable shifts in injury patterns during lockdown periods have been reported in several international studies [9,13,15]. Notably, however, these changes were transient and did not translate into long-term alterations in overall fracture epidemiology.

The absence of a sustained reduction in fracture incidence may be explained by adaptive behavioral responses. Although structured activities such as school and organized sports were restricted, children may have compensated by engaging in alternative forms of physical activity, including outdoor play and cycling. Such behavioral adaptations may have maintained an overall stable risk of injury despite shifts in the context of exposure.

Several limitations should be acknowledged. First, incidence rates were estimated based on the pediatric population of the catchment area rather than derived from precise population registry data. Second, fracture classification was performed by a single reviewer, precluding assessment of interobserver reliability. Third, referral pathways and treatment patterns could not be fully controlled, as multiple healthcare providers serve the study region; consequently, some cases may not have been captured.

In addition, the present study did not include a formal comparative analysis of treatment strategies between pre-pandemic and pandemic periods. While treatment modality (operative versus nonoperative) was recorded, the study was primarily designed as an ecological time-series analysis focusing on incidence patterns rather than individual-level treatment decisions. Therefore, the statistical framework was not optimized for subgroup comparisons at the patient level. Nevertheless, descriptive data suggest that the proportion of operative versus nonoperative treatment remained largely unchanged over time, indicating that the pandemic did not substantially alter treatment strategies in this cohort. Future studies using patient-level analytical approaches may provide further insight into potential changes in fracture severity and management during periods of societal disruption.

Finally, given the retrospective and exploratory design and the potential for unmeasured confounding, causal inferences regarding the relationship between COVID-19–related measures and fracture incidence remain limited despite the use of time-series regression techniques. Fracture classification was performed by a single reviewer without a formal assessment of interobserver reliability. This may introduce classification bias and limit confidence in the reported distribution of fracture types. Furthermore, complication rates could not be reliably assessed in the present study. Due to the retrospective design and the lack of standardized follow-up, complications such as re-displacement, secondary interventions, or delayed presentations may not have been consistently documented within our institutional dataset. In addition, some patients may have received follow-up care in external healthcare facilities, further limiting data completeness. As a result, any analysis of complication rates would be subject to substantial bias and was therefore not performed. Future studies incorporating prospective or longitudinal patient-level follow-up are needed to evaluate the impact of pandemic-related changes on fracture outcomes and complication rates. The restriction index assigned equal weight to all domains, which represents a simplification and may not fully reflect the relative importance of different measures. More complex weighting approaches were not applied due to the limited number of observations and the exploratory nature of the analysis.

The population denominator used to estimate incidence rates represents an approximation based on regional demographic assumptions. This approach does not account for migration, changes in population structure over time, or the possibility that not all injured children within the catchment area presented to the study center. Therefore, the reported incidence rates should be interpreted as estimated values rather than precise population-based measures.

Notwithstanding these limitations, this study provides comprehensive insight into pediatric fracture epidemiology across both pre-pandemic and pandemic periods. By explicitly accounting for seasonal variation and long-term trends, the analysis offers a more refined understanding of the impact of large-scale societal disruptions on injury patterns in children.

## 5. Conclusions

In conclusion, this study found no evidence of sustained changes in the incidence of pediatric forearm fractures during the COVID-19 pandemic. Although monthly fracture counts declined during the initial phase of the pandemic, these fluctuations were not independently associated with governmental restrictions after adjustment for seasonal variability and long-term temporal trends. Falls related to means of transportation represented the most frequent specific mechanism of injury. A better understanding of common injury mechanisms in pediatric populations may contribute to the development of targeted prevention strategies and improved long-term fracture risk reduction.

## Figures and Tables

**Table 1:** Distribution of fracture localization.

Fracture Localization	Absolute Numbers *n*	Percentage *n* (%)	Cumulative Percentage *n* (%)
Diaphyseal radius fracture	104	5.2%	5.2%
Diaphyseal ulna fracture	44	2.2%	7.3%
Diaphyseal forearm fracture	348	17.3%	24.6%
Metaphyseal radius fracture	788	39.1%	63.8%
Metaphyseal ulna fracture	6	0.3%	64.1%
Metaphyseal forearm fracture	394	19.6%	83.6%
Epiphysiolysis of the radius	314	15.6%	99.2%
Epiphysiolsis of the ulna	4	0.2%	99.4%
Epiphysiolysis of the forearm	4	0.2%	99.6%
Isolated bony avulsion of the radius/ulna	8	0.4%	100.0%

**Table 2:** Distribution of fracture types of the radius.

Fracture Type Radius	Absolute Numbers *n*	Percentage *n* (%)	Cumulative Percentage *n* (%)
Complete fracture	251	12.5%	15.4%
Greenstick fracture	290	14.4%	29.8%
Torus fracture	1037	51.5%	81.3%
Bowing fracture	26	1.3%	82.6%
Epiphysiolysis SH I	100	5.0%	87.5%
Epiphysiolysis SH II	210	10.4%	98.0%
Epiphysiolysis SH III	9	0.4%	98.4%
Epiphysiolysis SH IV	13	0.6%	99.1%
Epiphysiolysis SH V	2	0.1%	99.2%
Incomplete fracture	8	0.4%	99.6%
Comminuted fracture	6	0.3%	99.9%
Radial styloid fracture	2	0.1%	100.0%
Capsular avulsion fracture of the radius	1	0.0%	100,0%
Isolated ulna fracture (no radius fracture)	59	2.9%	2.9%
Total	2014	100.0%	

**Table 3:** Distribution of injury causes by age group.

Injury Causes	0–5 Years *n*	6–11 Years *n*	12–17 Years *n*	Total *n*
Fall/injury not specified/not reported	82	135	50	267
Fall/injury in private area (home/friends/family)	151	99	20	270
Fall/injury in kindergarten/school/after-school care center	28	123	78	229
Fall/injury outdoors/leisure time	79	104	32	215
Fall/injury during ball sports	8	121	115	244
Fall/injury involving means of transportation	25	198	75	298
Fall/injury during winter sports	1	58	44	103
Fall/injury at the playground	111	136	15	262
Fall/injury during various sports	5	28	24	57
Traffic accident	0	5	29	34
Fall from height	10	16	3	29

## Data Availability

The datasets analyzed during the current study are available from the corresponding author on reasonable request.

## Supplementary Materials

The following supporting information can be downloaded at: https://www.mdpi.com/article/10.3390/medicina62050966/s1, Table S1: Detailed criteria for scoring governmental restriction measures and construction of the composite restriction index.

## Abbreviations

The following abbreviations are used in this manuscript:

CI	Confidence interval
ESIN	Elastic stable intramedullary nailing
IQR	Interquartile range
IRR	Incidence rate ratio
K-wire	Kirschner wire
SD	Standard deviation
SH	Salter–Harris
