# Supplementary material for: The Impact of COVID-19-Related Restrictions on the Incidence of Diaphyseal and Distal Forearm Fractures: A Retrospective Analysis
Source: Medicina (Kaunas). 2026 May 15;62(5):966. doi: 10.3390/medicina62050966 (PMC13208516; doi:10.3390/medicina62050966)
Supplement: Supplementary file 1 [file medicina-62-00966-s001.zip › medicina-4239684-supplementary.pdf]

Month	Kindergarten/ Primary School Operations	Secondary School Operations	Organized Sports Activities	Curfew Regulations	Restriction Index	
March-20	0	0	0	0	0	0 = Complete Restriction
April-20	1	0	0	1	2	1 = Partial Restriction
May-20	2	2	1	2	7	2 = No Restriction
June-20	2	2	1	2	7	
July-20	2	2	1	2	7	
August-20	2	2	1	2	7	
September-20	2	2	2	2	8	
October-20	2	2	2	2	8	
November-20	0	0	0	0	0	
December-20	1	0	0	0	1	
January-21	1	2	1	1	5	
February-21	2	2	1	1	6	
March-21	2	2	1	1	6	
April-21	0	0	0	0	0	
May-21	2	2	1	1	6	
June-21	2	2	1	1	6	
July-21	2	2	1	2	7	
August-21	2	2	1	2	7	
September-21	2	2	2	2	8	
October-21	2	2	2	2	8	
November-21	2	2	0	0	4	
December-21	2	2	0	0	4	
January-22	2	2	2	2	8	
February-22	2	2	2	2	8	
March-22	2	2	2	2	8	
April-22	2	2	2	2	8	
May-22	2	2	2	2	8	
June-22	2	2	2	2	8	
July-22	2	2	2	2	8	
August-22	2	2	2	2	8	
September-22	2	2	2	2	8	
October-22	2	2	2	2	8	
November-22	2	2	2	2	8	
December-22	2	2	2	2	8	
January-23	2	2	2	2	8	
February-23	2	2	2	2	8	
March-23	2	2	2	2	8	
April-23	2	2	2	2	8	
May-23	2	2	2	2	8	
June-23	2	2	2	2	8	

Table S1: Detailed criteria for scoring governmental restriction measures and construction of the composite restriction index.
